# Trauma-informed conversational agents for mental health: understanding user perspectives and experiences

**DOI:** 10.3389/fdgth.2026.1797681

**Published:** 2026-06-10

**Authors:** Xintong Lu, Shion Guha, Rachel Pfafman, Jessica A. Pater, Courtney L. Washington, Fayika Farhat Nova

**Affiliations:** 1 Parkview Health, Parkview Research Center, Fort Wayne, IN, United States; 2 Department of Computer Science, University of Toronto, Toronto, ON, Canada; 3 Faculty of Information, University of Toronto, Toronto, ON, Canada; 4 Parkview Health, Parkview Behavioral Health Institute, Fort Wayne, IN, United States; 5 Psychological Science Department, Northern Michigan University, Marquette, MI, United States

**Keywords:** artificial intelligence, chatbots, conversational agents, human-AI interaction, mental health, trauma-informed care

## Abstract

**Introduction:**

Mental health conversational agents (MHCAs) offer scalable, accessible psychological support yet raise concerns about safety and appropriateness for trauma, exposed users. While trauma-informed care (TIC) principles, emphasizing safety, trust, empowerment, collaboration, peer support, and cultural sensitivity, are well-established in clinical practice, their application and user interpretation in chatbot contexts remain unexplored. This study aimed to explore (1) how users conceptualize trauma-informed care in the context of mental health chatbots and (2) what factors can help us predict whether users perceive their chatbot interaction as trauma-informed.

**Methods:**

A 59-item web-based survey (REDCap) assessed demographics, technology proficiency, adverse life experiences, chatbot usage, and TIC perceptions among 606 participants recruited via ClickWorker, social media, and MyChart. Exploratory and confirmatory factor analyses identified latent TIC domains. Multivariable logistic regression determined predictors of trauma-informed perception.

**Results:**

High satisfaction (84.5%) and trauma-informed perception (92.9%) were reported. Factor analyses validated a five-domain structure: Trust & Transparency, Data Safety, Empowerment, Peer Support, and Cultural Sensitivity (CFI = 0.978; RMSEA = 0.045). Trust (OR = 3.89, *p* = .001), Empowerment (OR = 1.97, *p* = .025), and Peer Support (OR = 1.73, *p* = .021) significantly predicted trauma-informed perception. Convenience-driven use (OR = 0.33, *p* = .041) and smartphone proficiency (OR = 0.38, *p* = .033) showed negative associations.

**Discussion:**

This study provides empirical foundation for measuring trauma-informed design in MHCAs. Trust-building, emotional validation, and peer connection significantly shape user perceptions. Findings emphasize the necessity of aligning AI-based mental health tools with trauma-informed principles to enhance therapeutic safety, credibility, and inclusivity across diverse user populations.

## 1 Introduction

Conversational agents (CAs), or chatbots, are AI-driven systems designed to simulate natural human conversation through text or voice interfaces [1]. In mental health contexts, these technologies are increasingly used to deliver psychoeducation, self-guided therapy modules, and supportive dialogue that mimics elements of traditional psychotherapy [2, 3]. Several studies suggest that mental health conversational agents (MHCAs) can improve emotional well-being, reduce symptoms of depression and anxiety, and enhance users’ self-efficacy and motivation to seek care [4–7]. These tools typically draw on established therapeutic models such as cognitive behavioral therapy (CBT), mindfulness, and positive psychology, and provide continuous access, anonymity, and convenience, which may be particularly attractive for individuals with unmet mental health needs [8–10]. As a result, commercial and research-based MHCAs such as Woebot, Wysa, and Youper have proliferated across digital marketplaces and health systems [8, 9, 11, 12].

Despite their growing popularity, MHCAs raise questions about safety, ethical design, and therapeutic efficacy, particularly for vulnerable users [13–16]. Reviews have noted that the evidence base remains mixed, with heterogeneous outcomes and high risk of bias in many studies [4, 10]. Mental health chatbots span a heterogeneous landscape of systems, from earlier rule-based and scripted conversational agents to contemporary large language model (LLM)-based generative AI systems such as those underpinning Woebot and Wysa [17, 18]. While earlier systems relied on predefined scripts and limited natural language understanding, generative AI systems introduce distinct challenges, including the potential for plausible but clinically inappropriate responses, inconsistent emotional attunement, and limited accountability [19, 20]. Empirical comparisons of LLM-generated responses with those of licensed therapists have found that chatbots tend toward directive, advice-giving communication rather than the Socratic, exploratory dialogue characteristic of effective psychotherapy, and are currently considered unsuitable as standalone therapeutic agents [20]. A scoping review of 101 studies further identified privacy and confidentiality (61% of articles) and safety and harm (52%) as the most frequently discussed ethical concerns in conversational AI mental health research [16].High-profile incidents, including reports of inappropriate responses to suicidal ideation or encouragement of harmful behaviors, have heightened public concern and exposed critical limitations in design and oversight across both rule-based and AI-generated conversational systems [21–23].

Trauma-informed care (TIC) has emerged as a foundational framework for addressing safety and ethical considerations in both clinical and digital mental health contexts. Originally proposed by the Substance Abuse and Mental Health Services Administration (SAMHSA), TIC is a strengths-based approach to service delivery that emphasizes understanding and responding to the widespread, disempowering effects of trauma [24–26]. Rather than functioning as a discrete treatment modality, TIC involves acknowledging the impact of trauma and intentionally responding in ways that support safety and avoid retraumatization [27]. The framework is structured around six core principles: (1) safety; (2) trustworthiness and transparency; (3) peer support; (4) collaboration and mutuality; (5) empowerment, voice, and choice; and (6) attention to cultural, historical, and gender issues [27]. Given that many individuals experience traumatic events throughout their lives, regardless of their diagnoses or presenting conditions, TIC has become a foundational approach in mental health care and has been extended beyond traditional clinical settings to domains such as trauma-informed telehealth and trauma-informed computing [28–34]. Critically, recent scholarship has raised concern that generative AI systems may actively increase the risk of technology-mediated trauma among vulnerable populations, underscoring the urgency of applying trauma-informed principles to AI-enabled health tools [28]. A recent scoping review of 118 studies systematically mapping TIC principles in digital technology design found that while safety, trustworthiness, and transparency were the most commonly referenced principles, a majority of studies invoked trauma-informed language without adherence to standardized frameworks, revealing a persistent gap between TIC aspiration and implementation in digital contexts [24]. These trauma-informed approaches aim to maximize physical, psychological, and emotional safety in all health care interactions, fostering opportunities for empowerment, control, and healing through safe, collaborative patient-clinician relationships [31, 34].

Recently, scholars in human-computer interaction and digital health have advocated integrating TIC into the design of digital platforms and AI-enabled technologies, giving rise to the concept of trauma-informed computing [29, 35–37]. Parallel developments in companion robot design have similarly demonstrated the feasibility of embedding TIC principles, including safety, trust, self-compassion, and self-efficacy, into AI system design for trauma-affected populations, providing an important proof of concept for trauma-informed AI development more broadly [25].A trauma-informed chatbot would not only protect user privacy and emotional safety, but also communicate its capabilities and limitations clearly, support user autonomy over how and when to engage, and avoid language or interaction patterns that may be experienced as coercive, dismissive, or triggering [29]. Despite growing advocacy for trauma-informed design, a critical gap remains: existing research has primarily approached TIC from a design or developer perspective, with limited empirical attention to end-user experiences [24, 33, 38]. In particular, there is no well-established, user-centered evidence base that systematically examines how individuals perceive and respond to TIC principles in MHCA interactions, nor which design elements most strongly influence perceptions of psychological safety, trust, and empowerment in automated contexts. The present study directly addresses this gap by empirically investigating end-user perceptions of trauma-informed care in MHCA interactions. Specifically, we examine:

**RQ1:** How do users conceptualize TIC principles in the context of mental health chatbots?
**RQ2:** What factors predict whether participants perceive their chatbot experience as trauma-informed?

By centering user experiences and expectations, this study aims to clarify what trauma-informed care looks like in MHCA interactions and to identify actionable features that support perceived emotional safety, trust, and autonomy. The findings provide insights for developers, clinicians, and researchers seeking to implement TIC in digital mental health tools and ensure that MHCAs serve users in an ethical, supportive, and trauma-sensitive manner.

## 2 Materials and methods

### 2.1 Survey design

This study was conducted as part of a larger federally funded research project and was approved by the institutional review board (IRB) of the Parkview Health. A structured, self-administered online questionnaire was developed and administered via REDCap to assess users’ experiences with MHCAs, their perceptions of trauma-informed chatbot characteristics, and their overall satisfaction with chatbot interactions.

Given the lack of established instruments that operationalize trauma-informed care principles through end-user perspectives specifically in the context of human–AI chatbot interactions, we developed an instrument by mapping SAMHSA’s TIC framework to measurable constructs. The items were developed through conceptual grounding in prior TIC and related HCI/healthcare literature, followed by iterative refinement based on expert feedback to improve clarity and relevance to chatbot experiences [27, 31, 39–41].

The questionnaire was developed as a self-designed instrument and refined with inputs from an expert in clinical psychology with expertise in TIC (the health system the authors are from), as well as feedback from human-centered design experts with experience in mental health contexts and AI ethics. These inputs helped improve the clarity and relevance of the items while ensuring alignment with trauma-informed principles. Likert-scale response formats were used to allow participants to indicate the degree of their experiences or agreement in a consistent and interpretable manner across constructs. Item wording was adapted to reflect user-facing experiences with chatbots rather than clinical or provider-oriented language. [26, 27, 34, 42].

The survey comprised four primary content sections:

**Demographics**, which captured participants’ gender, age, race/ethnicity, education level, and history of adverse life experiences;
**Technology Experience**, which assessed participants’ proficiency with smartphones, artificial intelligence, and smart devices, as well as their motivations for using mental health chatbots;
**Trauma-Informed Chatbot Characteristics and Experiences**, which evaluated user perceptions of TIC principles as reflected in chatbot design and interaction quality; and
**Satisfaction Level**, which measured overall satisfaction and perceived trauma-informed quality of the chatbot interaction.

The survey in total includes 59 items, including multiple-response (checkbox), Likert-scale (rated from 1 = not good/proficient/never to 5 = very good/proficient/always), binary (yes/no), and open-ended questions. To ensure data quality, the survey included one attention-check item and two hidden bot-detection questions designed to identify inattentive or automated responses. While participation in all questions was not mandatory and respondents could skip items at their discretion, they were required to provide responses to demographic items and multiple-choice questions related to TIC. Participants received $10 in compensation for survey completion. Full survey items are provided in Supplementary Table S1.

### 2.2 Recruitment

Participants were recruited between December 2024 and April 2025 through three primary channels: the crowdsourcing platform Clickworker (n=488), online social media platforms (n=113), and the MyChart patient communication portal (n=5), yielding a final eligible sample of 606 individuals [43]. Participation was voluntary, and each participant received $10 as compensation for their time.

Clickworker, a trusted crowdsourcing platform, was selected as the primary recruitment channel given its access to a large, diverse, and verified workforce spanning a broad range of educational backgrounds [43]. Crowdsourcing platforms, such as Clickworker, are widely used in human-computer interaction and digital health research to recruit diverse, scalable participant samples [44]. To address common data-quality concerns associated with crowdsourced samples, several safeguards were implemented, including explicit attention-check items (e.g., “To make sure you are paying attention, please select the third option for this question”), embedded bot-detection questions (e.g., “What is your favorite color?” and “What is your favorite ice cream flavor”), and post hoc exclusion of respondents who reported using tools that did not meet the definition of a mental health chatbot. Only participants who successfully passed all quality checks were included in the final analytic sample.

Social media recruitment was conducted via Twitter and LinkedIn, where study information and a survey link were publicly posted. The same data-quality safeguards applied to the crowdsourced sample were implemented for social media respondents.

Recruitment via MyChart targeted Indiana-based patients aged 18 years or older who had at least one clinical encounter within the behavioral health or psychiatry departments of the authors’ health system in the preceding 12 months and an active behavioral or psychiatric episode documented in their record. Although recruitment for MyChart spanned approximately eight weeks, the resulting subsample (n=5 participants) was small and is therefore not interpreted as representative of a clinical population, likely reflecting the sensitivity of trauma-related research topics and the well-documented reluctance of clinical populations to participate in such studies

The study was not designed to exclusively recruit trauma-affected or help-seeking clinical populations; instead, recruitment was intentionally broad to reflect the real-world diversity of individuals, both with and without trauma exposure, who engage with digital mental health tools. This approach aligns with an increasingly recognized perspective in human-computer interaction and mental health technology research that such heterogeneous user populations are a valid and important focus of study. Accordingly, the study is framed as exploratory and user-centered rather than intended for clinical generalization.

### 2.3 Participants

A total of 606 individuals constituted the final eligible analytic sample. Eligibility required participants to be: (1) aged 18 or older; (2) self-reporting a history of mental health concerns; (3) having used at least one mental health chatbot; and (4) able to read and understand English. The informed consent item was displayed only to individuals satisfying all four criteria. Those who failed eligibility screening, quality checks, or provided invalid chatbot responses were excluded.

Participants were instructed to reflect on their most recent or most frequently used mental health chatbot, acknowledging that perceptions may reflect aggregated experiences across heterogeneous platforms. This approach aligns with the study’s objective of assessing trauma-informed characteristics of mental health chatbots at a conceptual level, while acknowledging that platform-level variability may influence factor structure and outcome estimates.

Overall, 62.4% of participants were female, 37.5% were aged 25–34 years, 75.2% identified as White, and 30.2% held a bachelor’s degree. A majority reported a history of household dysfunction (71.3%), followed by experiences of various forms of abuse. Detailed demographic characteristics of the final analytic sample are presented in Table 1.

**Table 1:** Demographic characteristics of participants by recruitment source (*N* = 606).

Characteristics	Clickworker	Social media	MyChart	Overall
	(*N* = 488)	(*N* = 113)	(*N* = 5)	(*N* = 606)
Gender				
Female	326 (66.8%)	47 (41.6%)	5 (100.0%)	378 (62.4%)
Male	152 (31.1%)	65 (57.5%)	0 (0.0%)	217 (35.8%)
Nonbinary	8 (1.6%)	0 (0.0%)	0 (0.0%)	8 (1.3%)
Transgender	1 (0.2%)	1 (0.9%)	0 (0.0%)	2 (0.3%)
Prefer not to say	1 (0.2%)	0 (0.0%)	0 (0.0%)	1 (0.2%)
Age group				
18–24 years	37 (7.6%)	12 (10.6%)	0 (0.0%)	49 (8.1%)
25–34 years	174 (35.7%)	51 (45.1%)	2 (40.0%)	227 (37.5%)
35–44 years	183 (37.5%)	41 (36.3%)	1 (20.0%)	225 (37.1%)
45–54 years	87 (17.8%)	9 (8.0%)	2 (40.0%)	98 (16.2%)
55–64 years	7 (1.4%)	0 (0.0%)	0 (0.0%)	7 (1.2%)
65 years or older	0 (0.0%)	0 (0.0%)	0 (0.0%)	0 (0.0%)
Race				
White	373 (76.4%)	79 (69.9%)	4 (80.0%)	456 (75.2%)
Black	77 (15.8%)	23 (20.4%)	0 (0.0%)	100 (16.5%)
Asian	37 (7.6%)	3 (2.7%)	0 (0.0%)	40 (6.6%)
American Indian	15 (3.1%)	7 (6.2%)	0 (0.0%)	22 (3.6%)
Pacific Islander	2 (0.4%)	2 (1.8%)	0 (0.0%)	4 (0.7%)
Other	18 (3.7%)	0 (0.0%)	1 (20.0%)	19 (3.1%)
Missing	1 (0.2%)	0 (0.0%)	0 (0.0%)	1 (0.2%)
Adverse life experiences				
Abuse	323 (66.2%)	53 (46.9%)	5 (100.0%)	381 (62.9%)
Adoption	19 (3.9%)	6 (5.3%)	1 (20.0%)	26 (4.3%)
Child services involvement	79 (16.2%)	7 (6.2%)	2 (40.0%)	88 (14.5%)
Community adversity	198 (40.6%)	39 (34.5%)	3 (60.0%)	240 (39.6%)
Household dysfunction	372 (76.2%)	55 (48.7%)	5 (100.0%)	432 (71.3%)
Neglect	234 (48.0%)	50 (44.2%)	4 (80.0%)	288 (47.5%)
Education level				
Less than high school	12 (2.5%)	0 (0.0%)	0 (0.0%)	12 (2.0%)
High school or GED	94 (19.3%)	3 (2.7%)	0 (0.0%)	97 (16.0%)
Some college (no degree)	129 (26.4%)	5 (4.4%)	2 (40.0%)	136 (22.4%)
Technical certification	25 (5.1%)	3 (2.7%)	0 (0.0%)	28 (4.6%)
Associate degree	55 (11.3%)	26 (23.0%)	2 (40.0%)	83 (13.7%)
Bachelor’s degree	127 (26.0%)	55 (48.7%)	1 (20.0%)	183 (30.2%)
Master’s degree	41 (8.4%)	14 (12.4%)	0 (0.0%)	55 (9.1%)
Doctoral degree	2 (0.4%)	7 (6.2%)	0 (0.0%)	9 (1.5%)
Prefer not to say	2 (0.4%)	0 (0.0%)	0 (0.0%)	2 (0.3%)
Missing	1 (0.2%)	0 (0.0%)	0 (0.0%)	1 (0.2%)

### 2.4 Data analysis

Qualitative data from open-ended survey questions were analyzed using inductive reflexive thematic analysis to identify recurring patterns and user-defined concepts related to trauma-informed chatbot experiences. Reflexivity was maintained throughout by the first and last authors to ensure transparency in interpretive decision-making [45].

Quantitative survey data, including multiple-response and Likert-scale items, were analyzed using a combination of descriptive and inferential statistical methods. Descriptive statistics were first computed to summarize participant characteristics, technology use patterns, and overall perceptions of trauma-informed care. Exploratory factor analysis (EFA) was then conducted to identify latent domains underlying participants’ conceptualization of trauma-informed chatbot characteristics. Confirmatory factor analysis (CFA) was subsequently performed to validate the factor structure and evaluate model fit. Finally, multivariable logistic regression models were fitted to identify factors associated with participants’ perceptions of chatbot interactions as trauma-informed.

**Figure 1:**
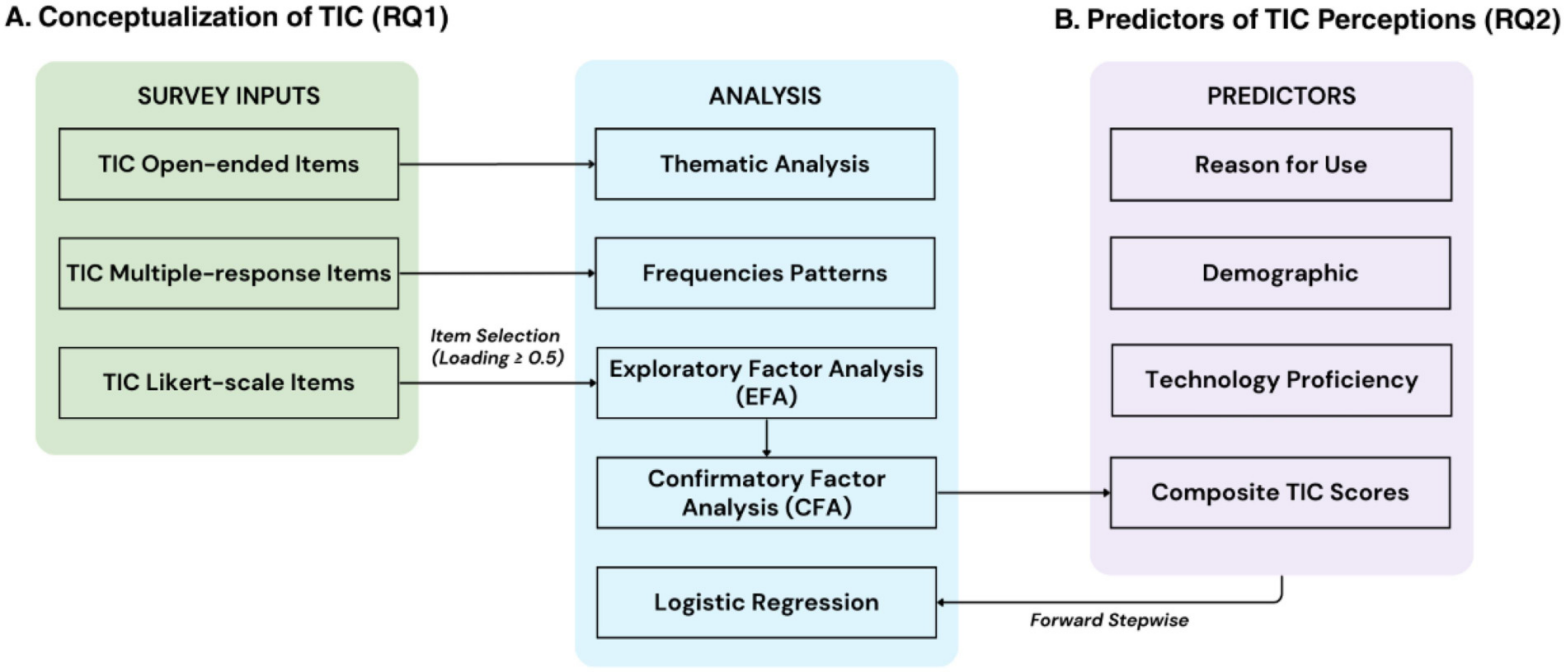
Overview of the analytic workflow used to address the study research questions.

All statistical analyses were conducted using R (version 4.4.3) and relevant statistical packages. An overview of the analytic workflow used to address the study research questions is presented in Figure 1.

#### 2.4.1 Descriptive analysis

Descriptive statistics were computed to summarize participants’ overall satisfaction, technology proficiency, chatbot use and motivations, as well as multiple-response items on TIC perceptions. Frequency distributions and proportions were reported for categorical and binary variables, while means and standard deviations were calculated for continuous and ordinal variables.

#### 2.4.2 Factor analysis

To identify the underlying structure of TIC perceptions, an EFA was conducted on the full set of Likert-scale items from the TIC section of the survey. EFA was chosen given the exploratory aim of identifying latent constructs underlying participant responses, as no established factor structure was assumed. Prior to analysis, all variables were assessed for missing data. The average item-level missing rate was <2%. To preserve the integrity of the original responses, no imputation was performed; instead, cases with missing data were excluded listwise from the analysis. EFA was conducted using maximum likelihood estimation on the correlation matrix, with an oblimin rotation applied to account for potential correlations among factors. The number of factors retained was guided by parallel analysis. A stepwise item reduction procedure was applied, sequentially removing the item with the lowest standardized loading at each iteration until all retained items demonstrated primary factor loadings ≥0.50 to ensure strong associations with the extracted factors. Following item selection, the theoretical framework was refereed to ensure retained items remained conceptually coherent. CFA was subsequently conducted to test the fit of the TIC model identified in the EFA phase. CFA models were evaluated using standard goodness-of-fit indices, including the Comparative Fit Index (CFI), Tucker-Lewis Index (TLI), Root Mean Square Error of Approximation (RMSEA), and Standardized Root Mean Square Residual (SRMR).

Because EFA and CFA were performed on the same sample, results are exploratory and descriptive, intended to examine internal structure rather than definitive model validation. Nevertheless, the combination of theory-informed item development, stringent loading thresholds, oblique rotation, and multiple fit criteria supports the internal coherence and preliminary construct validity of the proposed TIC factor structure. Future research should replicate and validate this model in independent, clinically representative samples.

#### 2.4.3 Logistic regression model

Logistic regression analyses were conducted to examine predictors of whether participants perceived their chatbot experience as trauma-informed. Composite scores were first calculated from the validated CFA structure, with items loaded on the same latent factor averaged to form summary scores for each dimension. These composite scores were merged with demographic variables and technology proficiency data to serve as predictors in a multivariable logistic regression model. A forward stepwise selection procedure was used to identify the most parsimonious model. Final model estimates are reported as odds ratios (ORs) with corresponding p-values and 95% confidence intervals (CIs), providing insight into which chatbot features are most strongly associated with positive TIC perceptions.

## 3 Result

### 3.1 General descriptive statistics

#### 3.1.1 Overall satisfaction

A total of 606 surveys were included in analysis. Overall, 92.9% of respondents perceived the chatbot as trauma-informed, with similarly high endorsement across recruitment sources (Clickworker: 91.6%, social media: 100.0%, MyChart: 60.0%). Likewise, 92.6% of participants reported they would recommend the chatbot to others with a trauma history (Clickworker: 91.0%, social media: 100.0%, MyChart: 80.0%). Satisfaction ratings further supported chatbot acceptability. On a five-point scale ranging from “very dissatisfied” to “very satisfied,” most participants expressed a positive experience, with 84.5% selecting the top two satisfaction levels (48.0% rated it as satisfied and 36.5% as extremely satisfied). Lower satisfaction ratings were rare, with only 2.9% of respondents expressing dissatisfaction. These patterns were generally consistent across all recruitment sources.

#### 3.1.2 Technology experience

Participants demonstrated generally high levels of self-reported proficiency across all six technology domains: smartphone use, internet browsing, AI tools, computer use, social media, and smart home devices. Among these, smartphone use (M = 4.73, SD = 0.54) and internet browsing (M = 4.71, SD = 0.56) proficiencies were rated highest across the full sample. In contrast, proficiency in smart home device use was the lowest overall (M = 3.99, SD = 1.02), reflecting broader variability in exposure to and use of emerging technologies. Clickworker respondents reported the highest average proficiency overall (M = 4.44, SD = 0.82), followed by social media participants (M = 4.37, SD = 0.86). MyChart participants (M = 4.10, SD = 0.71) had slightly lower average scores across most domains. Despite these differences, mean scores remained high, indicating strong baseline digital literacy among participants. The details can be seen in Supplementary Figure S1.

#### 3.1.3 Chatbot use and motivations

**Figure 2:**
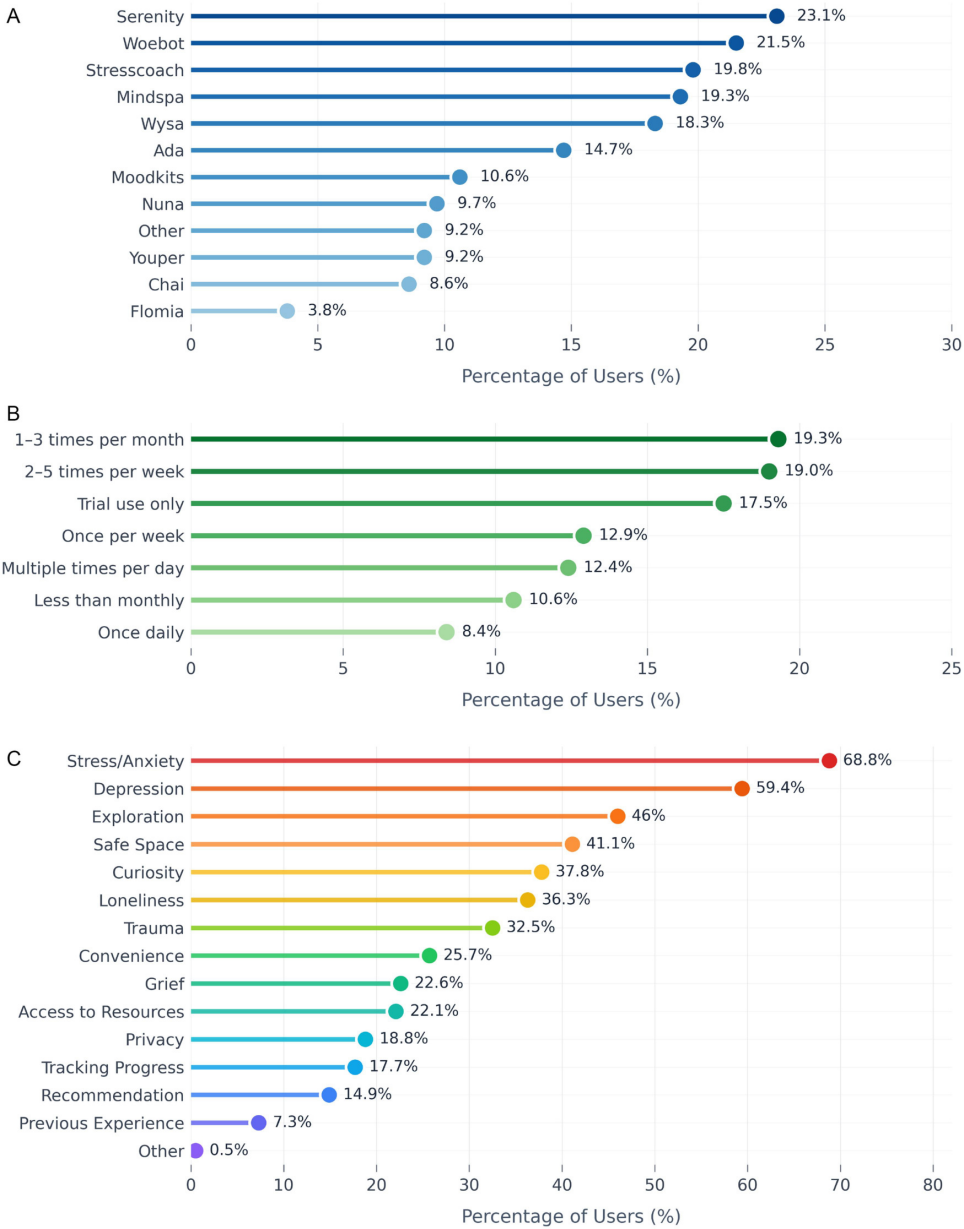
Patterns of mental health chatbot use among participants. **(A)** Distribution of mental health applications used. **(B)** Self-reported frequency of application usage. **(C)** Primary reasons for using mental health applications. Respondents could select multiple options; percentages reflect the proportion of the total sample.

Participants reported varied engagement with mental health chatbots in terms of both frequency and platform type. The results are presented in Figure 2. Most used the chatbot 1–3 times per month (19.3%) or 2–5 times per week (19.0%), while 17.5% reported trying it only once. Daily use was less common (8.4%), indicating that chatbots may be serving as intermittent support tools rather than daily companions for most users. For platforms, the most frequently used chatbots were Serenity (23.1%), Woebot (21.5%), and Stresscoach (19.8%). Participants reported diverse motivations for engaging with mental health chatbots. The most endorsed reasons were related to emotional distress, including stress or anxiety (68.8%) and depression (59.4%). A substantial proportion also reported exploratory motives such as self-guided exploration (46.0%) and curiosity (37.8%). The desire for a psychologically safe space was also a prevalent motivation (41.1%), ranking higher than expected clinical drivers such as trauma (32.5%) or loneliness (36.3%). Less commonly selected reasons included privacy concerns, recommendations, and previous experience, each under 20%.

### 3.2 RQ1: conceptualization of TIC principles

#### 3.2.1 Users perceptions across six core dimensions

To explore how users conceptualized trauma-informed care (TIC) in the context of mental health chatbots, participants’ responses to multiple-response checkbox items aligned with the six core TIC principles were summarized. Both individual feature frequencies and co-occurrence patterns were examined to characterize how users collectively defined a trauma-informed chatbot.

**Safety** was primarily defined as the ability to interact with the chatbot without fear or judgment. Emotional safety was the most frequently selected feature within this domain (86.7%), highlighting the importance of psychological security in chatbot interactions. The most common feature combination (28.9%) included emotional safety, physical safety, and digital safety, indicating a holistic view of safety spanning psychological and technical dimensions.

**Trust and transparency** centered on ensuring that users feel respected through open and clear communication. Information confidentiality emerged as the highest-priority feature (82.3%), underscoring strong concerns regarding privacy. A frequently occurring combination (26.7%) included transparency, tailored responses, validation of experiences, and data care, suggesting that trust is closely linked to both personalization and responsible data handling.

**Empowerment** emphasized user autonomy and support. Guide conversation was the most frequently endorsed feature (73.1%), underscoring the importance of user-directed dialogue. The most common combination (20.8%) included sharing control, no forced choice, expression options, hopeful messaging, and sense of agency, reflecting preferences for tools that foster autonomy and emotional encouragement.

**Collaboration** reflected partnership and shared decision-making. Respect choices was the most frequently selected design element (72.6%). A commonly endorsed combination (38.8%) included goal setting, shared goals, and respect choices, suggesting that participants valued mutuality and co-creation of a therapeutic path.

**Peer support** focused on connection with others who have similar lived experiences. Ask for support was the most frequently selected feature (73.1%), highlighting the importance of self-initiated outreach. The most common configuration (19.8%) included offer support, support available, relevant connections, and virtual or in-person support, indicating interest in accessible and meaningful peer engagement.

**Cultural sensitivity** emphasized inclusivity and respect for diverse identities. Nonjudgmental was the most frequently endorsed feature (79.4%), reflecting a strong desire for acceptance. The most common feature combination (23.8%) included sensitive to identity, avoids offense, avoids triggers, validates experience, shows interest, and culturally aligned, highlighting preferences for chatbots that are affirming, inclusive, and mindful of cultural and individual differences.

Full item-level response distributions are presented in Supplementary Figure S2, and detailed item wording is provided in Supplementary Table S2.

#### 3.2.2 Uncovering latent constructs of trauma-informed experience

Participants’ conceptualization of trauma-informed chatbot design was further explored through Likert-scale ratings, in which they indicated their level of agreement (strongly disagree to strongly agree) with statements reflecting the six TIC principles. This measure captured the extent to which users endorsed statements such as, “My chatbot is sensitive to my concerns, identity, and/or historical experiences.” Overall, responses reflected favorable perceptions: 75.2% of participants selected a positive response (Agree: 35.5%; Strongly Agree: 39.7%), 16.7% were neutral, and only a small minority expressed disagreement (Disagree: 5.6%; Strongly Disagree: 2.6%). Item-level response distributions are presented in Supplementary Figure S3.

While these descriptive findings suggest broadly positive user perceptions, prior HCI research cautions that high endorsement alone does not reveal how users cognitively organize or differentiate experiential constructs in human–AI interaction [46]. Accordingly, we conducted an EFA to examine whether participants’ responses aligned with the six theoretically specified TIC principles or reflected an alternative latent structure shaped by the interactional affordances of chatbot systems.

Parallel analysis supported a five-factor solution. The final EFA model retained 18 items and explained 66% of the total variance, with individual factors accounting for 7% to 20% of the explained variance. Based on item content and theoretical coherence, the five factors were labeled *Peer Support*, *Trust and Transparency*, *Data Safety*, *Empowerment*, and *Cultural Sensitivity*. These factors represent the most salient trauma-informed dimensions as experienced by users during real-world chatbot interactions.

Four of the five factors mapped closely to SAMHSA TIC principles. *Peer Support* reflected users’ desire for connection to others with shared lived experiences, consistent with prior findings that perceived social connectedness, whether direct or mediated, is central to supportive digital mental health experiences [47]. *Trust and Transparency* encompassed perceptions of reliability, expectation alignment, and responsiveness, echoing HCI literature demonstrating that trust in conversational agents is shaped by predictability and clarity rather than anthropomorphism alone [48]. *Empowerment* captured feelings of agency, autonomy, and emotional self-efficacy, aligning with self-determination theory and prior work on empowering AI design [49, 50]. *Cultural Sensitivity* reflected inclusive language use and avoidance of triggering content, consistent with research highlighting linguistic safety as a key determinant of perceived care quality in digital mental health tools [51]. *Data safety* emerged as a distinct latent construct, encompassing items related to digital safety, privacy being respected, and careful handling of personal data, rather than as part of a broad, undifferentiated safety factor.

The SAMHSA principle of *Collaboration* did not emerge as a distinct factor. Instead, collaboration-related items loaded onto adjacent domains, particularly *Empowerment* and *Trust and Transparency*. This pattern suggests that users may conceptualize collaboration with chatbots less as reciprocal partnership and more as feeling supported, respected, and enabled. Factor correlations ranged from 0.31 to 0.68, indicating related but distinct constructs, and mean item complexity was low (1.1), supporting factor interpretability. Model fit was acceptable (RMSR = 0.02; $\chi^{2}=146.61$, df=73, p<.001), and internal consistency was strong across factors (Cronbach’s α=0.77–0.92). Detailed factor loadings and communalities are reported in Supplementary Table S3.

#### 3.2.3 Validating the factor structure of trauma-informed experience

To evaluate the adequacy of the factor structure identified through EFA, a CFA was conducted using maximum likelihood estimation. The five-factor, 18-item model demonstrated excellent fit to the data (CFI = 0.978; TLI = 0.973; RMSEA = 0.045, 90% CI: 0.037–0.052; SRMR = 0.033). All standardized factor loadings were statistically significant (p<.001) and ranged from 0.70 to 0.88, indicating strong associations between observed indicators and their respective latent constructs.

Among the five latent domains confirmed through CFA, Peer Support emerged as a particularly cohesive and robust construct, with nearly all items demonstrating strong factor loadings. Most notably, the item “Q42. Support Available: My mental health chatbot offers opportunities for peer support and community connections as part of my mental health management” exhibited the highest standardized loading in the entire model (0.88), establishing it as a central indicator of this domain.

Within the Trust and Transparency domain, the item “Q22. Meets Expectations: My chatbot responds to my experiences in a way that meets my expectations” had the highest loading (0.81), highlighting its critical role in shaping user perceptions of trustworthiness and responsiveness. In the Data Safety domain, “Q15. Privacy Respected: My mental health chatbot respects my privacy and confidentiality during interactions” demonstrated the strongest loading (0.83), affirming that users prioritize digital privacy and data protection as foundational to feeling safe in chatbot interactions. For Empowerment, the leading item was “Q34. Sense of Agency: My chatbot helps me believe that I have agency/ability to change things in my life,” which yielded a loading of 0.88, underscoring the importance of fostering personal autonomy and emotional self-efficacy. Lastly, within Cultural Sensitivity, “Q51. Avoids Triggers: My chatbot avoids words or phrases that may trigger me” showed the strongest association (0.81), emphasizing users’ sensitivity to emotionally safe language.

**Figure 3:**
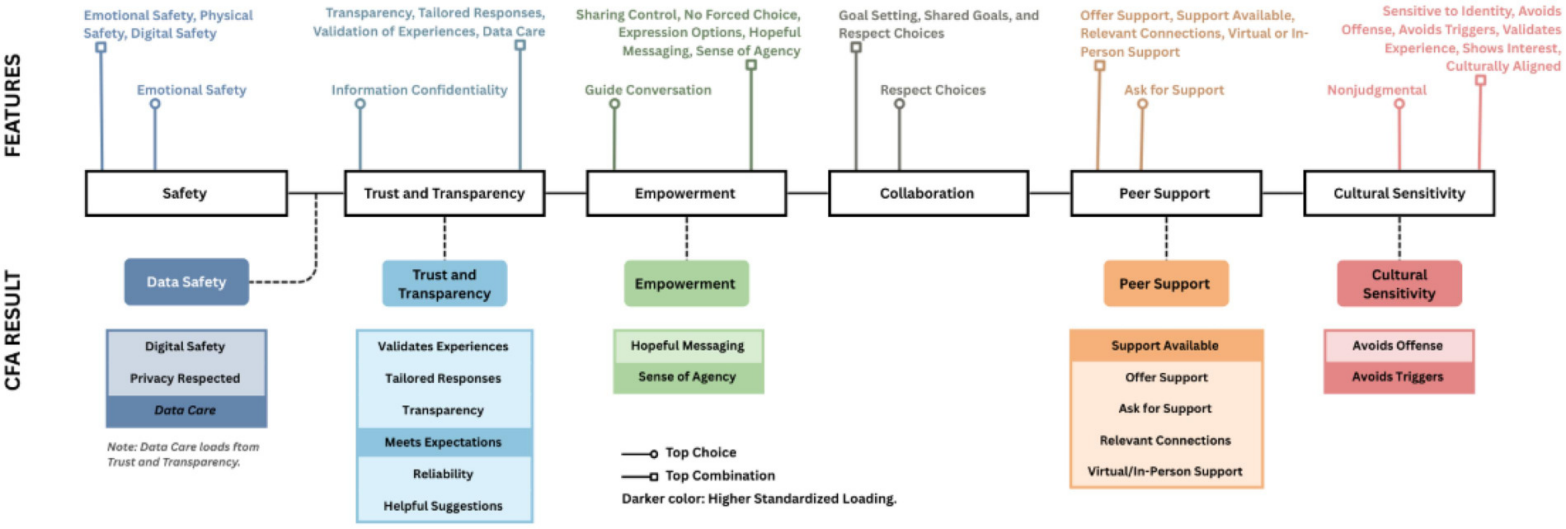
Summary of descriptive and confirmatory factor findings on trauma-informed chatbot features. The top portion summarizes participants’ most frequently endorsed features (circle endpoints) and commonly co-selected combinations (square endpoints) across the original six trauma-informed care principles. The bottom portion displays the final five-factor structure derived from confirmatory factor analysis, with darker colors indicating items with higher standardized factor loadings.

**Figure 4:**
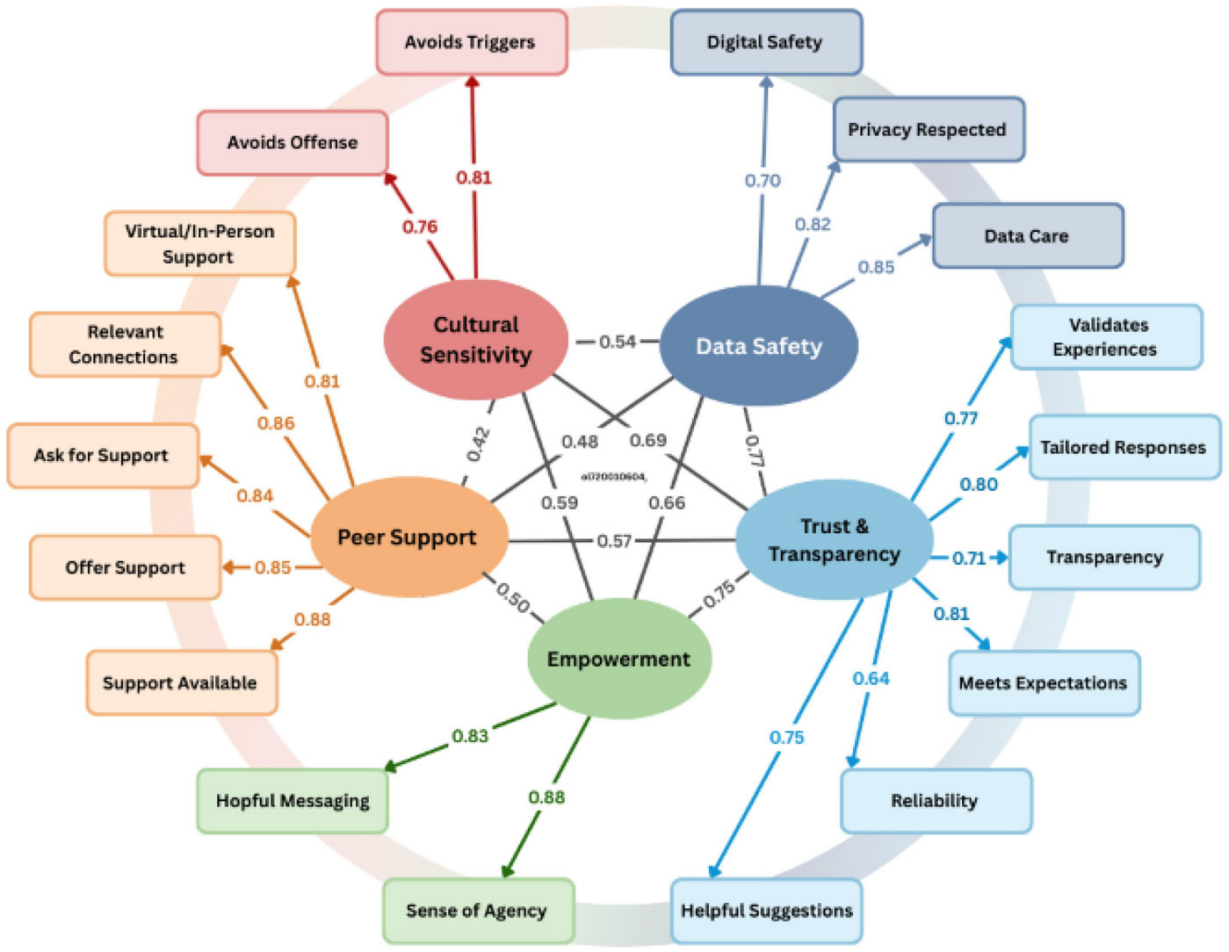
Factor structure with standardized loadings for the Confirmatory Factor Analysis model of trauma-informed chatbot perception.**TrS**: Trust; **Sft**: Safety; **Emp**: Empowerment;**PrS**: Peer Support; **SnS**: Cultural Sensitivity. Bar widths represent the magnitude of standardized loadings, with values above 0.80 indicating particularly strong associations between items and their respective latent constructs.

Figure 3 presents a synthesized summary of findings, integrating both the descriptive checkbox responses and the CFA results. The detailed standardized loadings for each item within the CFA model are presented in Figure 4 and Supplementary Table S4, supporting the scale’s construct validity and further clarifying item-factor relationships.

#### 3.2.4 Qualitative evidence supporting the five-factor structure

Qualitative findings further supported the five-factor structure identified through EFA and CFA by illustrating how users experienced each TIC domain in practice. Across domains, participants’ narratives provided concrete examples of how abstract TIC principles were enacted during chatbot interactions, thereby reinforcing the quantitative findings.

Within the **Trust and Transparency** domain, participants consistently emphasized the importance of emotional validation and responsive communication. Users described feeling understood by the chatbot, noting that it would “listen and understand,” and that “I trust it more because it feels like I’m talking with a caring and concerned friend, not a chatbot that just gives basic random responses.” These accounts highlight how perceived empathy and tailored responses foster trustworthiness in automated mental health support.

**Data Safety** was described as a holistic construct encompassing anonymity, immediacy of access, and digital protections. Several participants identified anonymity as central to their sense of safety, with one noting that “the main thing that helps me feel safe” was the ability to interact without revealing personal identity. Others emphasized the importance of data protection, explaining that “knowing my conversations are secure and private makes me feel safer.” Together, these responses illustrate how technical safeguards contribute to users’ perceptions of safety.

Experiences related to **Empowerment** centered on agency, encouragement, and goal support. Participants expressed appreciation for features that promoted self-direction and motivation, with one reporting that “consistent check-ins help keep me on track with my goals.” These reflections align with the quantitative findings, underscoring the role of empowerment in shaping trauma-informed perceptions.

Responses regarding **Peer Support** were more heterogeneous. While some participants preferred private, one-on-one chatbot interactions, others expressed interest in shared experiences, noting that “peer-to-peer interactions… would be appreciated sometimes.” This variability suggests that peer support is valued selectively and may benefit from flexible, opt-in design approaches.

Finally, **Cultural Sensitivity** emerged as a critical dimension of inclusion and respect. Participants highlighted the importance of consent, nonjudgmental language, and attentiveness to emotional boundaries, stating that “the bot does check in to see if I am comfortable” and “asks permission to discuss a sensitive topic further.” These accounts emphasize users’ expectations for culturally and emotionally attuned communication.

### 3.3 RQ2: identifying predictors for chatbot being trauma-informed

**Figure 5:**
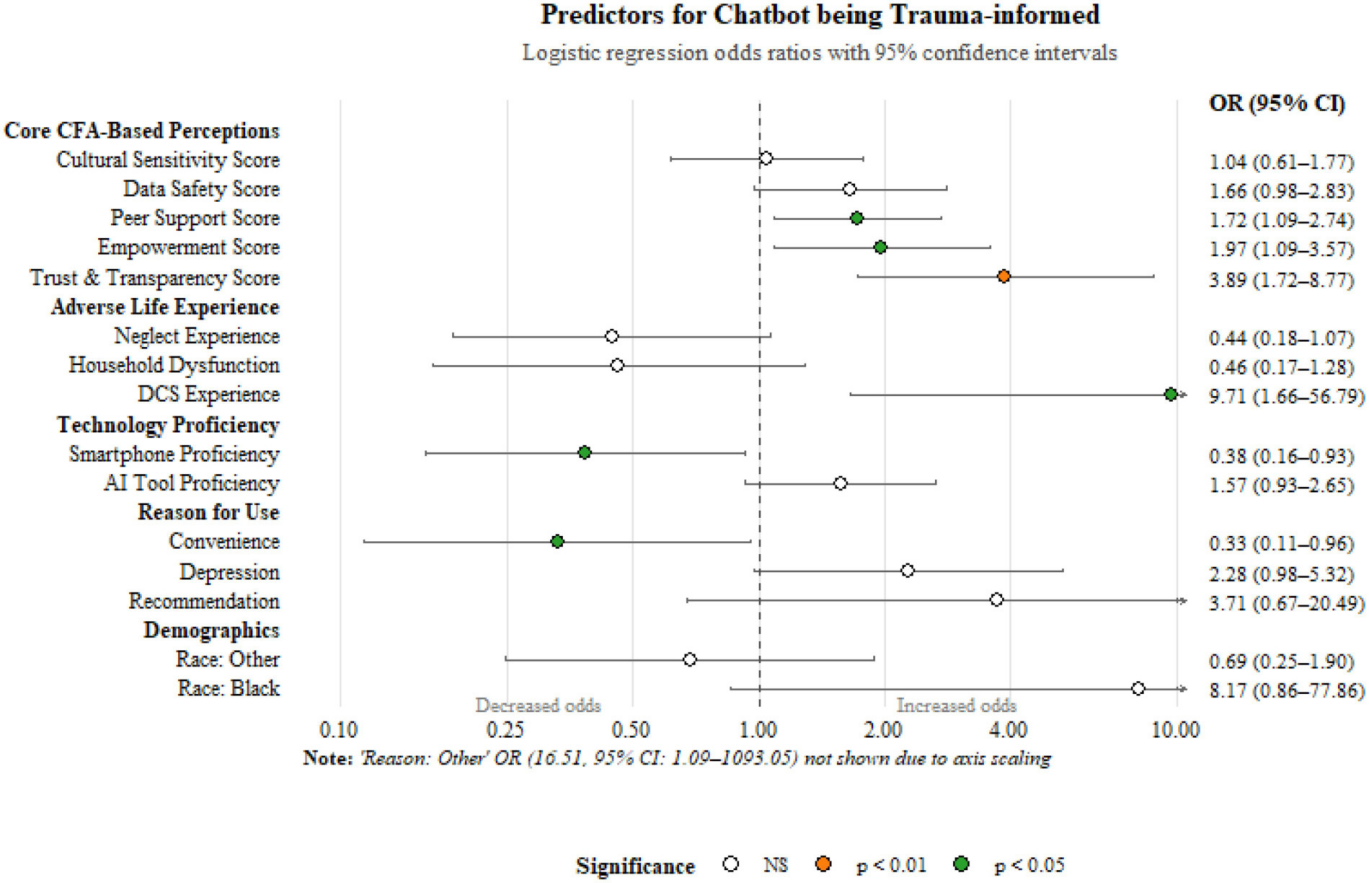
Multivariable logistic regression results showing predictors of perceived trauma-informed chatbot interaction. Estimates are presented as odds ratios with 90% confidence intervals and corresponding *p*-values.

To examine factors associated with perceiving a mental health chatbot as trauma-informed, we fitted a multivariable logistic regression model. Composite scores for the five CFA-derived dimensions were first entered into a baseline model. Forward stepwise selection was subsequently applied to identify a parsimonious set of predictors. Candidate variable *reason: others* were excluded from the final model due to estimation instability (e.g., near-infinite standard errors), driven by sparse category endorsement and small subgroup sizes. The final model results are summarized in Figure 5.

#### 3.3.1 Chatbot design features

Among design-related predictors, perceived *Trust* emerged as the strongest and most stable correlate of trauma-informed perception. Higher trust scores were associated with substantially increased odds of perceiving the chatbot as trauma-informed (OR = 3.89, 95% CI: 1.73–8.77, *p* = .001). *Peer Support* (OR = 1.73, 95% CI: 1.09–2.74, *p* = .021) and *Empowerment* (OR = 1.97, 95% CI: 1.09–3.57, *p* = .025) were also positively associated with trauma-informed perception, reinforcing the importance of relational responsiveness and autonomy-supportive features in shaping users’ evaluations of chatbot interactions.

#### 3.3.2 User characteristics

Several user-level characteristics further differentiated trauma-informed perceptions. Participants who reported *prior involvement with the Department of Child Services* exhibited markedly higher odds of perceiving the chatbot as trauma-informed (OR = 9.71, 95% CI: 1.66–56.79, *p* = .012). Although confidence intervals were wide, reflecting limited subgroup size, this finding suggests that individuals with lived experience of formal care systems may be particularly attuned to trauma-aligned design features.

In contrast, two predictors were negatively associated with trauma-informed perception. Users who cited *convenience* as their primary motivation for chatbot use had significantly lower odds of perceiving the chatbot as trauma-informed (OR = 0.33, 95% CI: 0.11–0.96, *p* = .041). Similarly, higher self-rated *smartphone proficiency* was inversely associated with trauma-informed perception (OR = 0.38, 95% CI: 0.16–0.93, *p* = .033). These associations may reflect differing evaluative standards among highly digitally proficient users, who may prioritize efficiency, performance, and usability over relational or emotionally supportive features, and therefore be less responsive to trauma-informed design cues.

## 4 Discussion

Our study offers a rigorous empirical foundation for understanding how users interpret, experience, and evaluate TIC features in mental health chatbot interactions. By integrating descriptive pattern analysis, confirmatory factor analysis, and predictive modeling, we elucidate both the conceptual structure of TIC as perceived by end-users and the key elements that drive trauma-informed appraisals.

Overall, users overwhelmingly perceived the chatbot as embodying TIC characteristics, with more than 90% endorsing their experience as trauma-informed alongside similarly high rates of satisfaction and recommendation. Importantly, participants did not interpret TIC as a monolithic construct. Instead, both quantitative and qualitative findings revealed a nuanced understanding spanning emotional safety, privacy, identity validation, agency, and peer connection. These themes align with existing TIC theory while contributing new empirical evidence to its application in digital mental health contexts [26, 27, 34, 42]. Rather than viewing TIC as a single construct, participants emphasized key design features that shaped how they judged the chatbot’s empathy, safety, and trustworthiness. Emotional Safety and Information Confidentiality were most valued, confirming that freedom from fear, judgment, and privacy concerns defines psychological safety in digital care [2, 19, 20, 52]. Features that allowed users to guide conversations and receive hopeful, personalized responses fostered empowerment and a sense of agency [52, 53]. Respect for user choices and adaptive feedback conveyed collaboration and mutuality without explicit co-decision-making [29, 54, 55]. Opportunities for self-initiated peer connection further enhanced perceived support and belonging [47, 56]. Finally, a nonjudgmental tone and cultural awareness reinforced inclusivity and emotional comfort across backgrounds [39]. Together, these design elements illustrate that users interpret trauma-informed chatbots through an integrated lens of emotional security, autonomy, relational responsiveness, and cultural respect.

Our factor analysis results yielded a five-factor structure comprising Trust and Transparency, Data Safety, Empowerment, Peer Support, and Cultural Sensitivity. The factor analysis revealed a distinct Data Safety latent factor, which suggests that, for our population, safety was primarily understood and evaluated through the lens of data protection and privacy, rather than a generalized sense of emotional or interpersonal safety. In the context of mental health chatbots, this distinction indicates that assurances about how user data are stored, protected, and used may be especially salient for individuals with lived experiences of vulnerability or trauma and may meaningfully shape their perceptions of whether an interaction feels safe. The absence of a distinct Collaboration factor, despite its theoretical salience, suggests that collaborative elements (e.g., shared decision-making) may be implicitly embedded within other domains. Qualitative accounts reinforced this interpretation: several participants emphasized valuing “consistent feedback” and the sense that responses were “tailored to me,” underscoring collaboration as an integrated rather than standalone dimension.

Trust and Transparency emerged as the most robust driver of trauma-informed perception, especially in relation to confidentiality and expectation management. Emotional validation played a central role in cultivating trust, as reflected in both model estimates and user narratives. Participants consistently described the importance of feeling “listened to and understood” and noted that empathetic language made them feel “like I’m talking with a caring and concerned friend, not just a chatbot.” Such validation highlights how relational design elements underpin trust and drive trauma-informed evaluations. This finding aligns with prior research demonstrating that relational authenticity and transparent communication are essential for trust-building in digital mental health tools [19, 20].

Empowerment and Peer Support also proved to be critical dimensions for shaping trauma-informed perceptions. Users who felt supported in decision-making or had access to community-based resources were significantly more likely to view the chatbot as trauma-informed, echoing findings by Potts et al., who observed that multilingual chatbots offering user-driven dialogue improved well-being and engagement [53]. Similarly, participants valued access to supportive, community-oriented features, paralleling Beilharz et al., who emphasized that peer-based and family-linked chatbot components enhanced feelings of connection and emotional safety among adolescents [56]. This aligns with core TIC principles that emphasize relational healing and shared agency as foundational to care [4, 27].

Individual differences further shaped TIC perceptions. Participants in our study with a history of involvement with child protective or social services had significantly higher odds of perceiving the chatbot as trauma-informed.This finding may be understood through the lens of system-impacted populations’ unique relationships with institutional support and technology. Research on youth with foster care or child welfare involvement has documented that such experiences often lead to distinctive patterns of help-seeking behavior and trust formation [57–60]. Youth in foster care frequently experience multiple placements and disrupted relationships, which can profoundly shape their expectations and evaluations of new support systems, whether human or technological [58, 60–63]. Studies examining digital health adoption among foster youth suggest that this population approaches technology-mediated support with both strategic caution and openness, particularly valuing features that signal safety, autonomy, and non-coercive care [59, 60, 64]. These individuals often develop what might be termed “system literacy”: a heightened ability to discern whether systems, including digital ones, are designed with their safety and dignity in mind [61–63]. Prior work has also shown that young people who have experienced foster care, adoption, or removal from biological families may be particularly attuned to relational qualities in both human and technological interactions, given their histories of disrupted attachments and mandated institutional relationships [58, 65–69].

Consistent with this interpretation, research on relational agents has shown that users experiencing higher emotional vulnerability, including depressive symptoms or psychological distress, tend to form stronger therapeutic alliances with conversational systems [70, 71] For individuals with child welfare histories, this effect may be compounded: digital agents may represent a form of support perceived as less surveilling, less coercive, and more user-controlled than traditional institutional services [57, 61, 63, 72]. These studies suggest that conversational agents may be experienced as emotionally safer or less threatening than human interlocutors, in part because they offer predictable, nonjudgmental interactions and reduce fears of stigma or social evaluation [61, 62, 72]. Youth in foster care often report mistrust of service providers and concerns about stigma when seeking mental health support , barriers that may be attenuated in interactions with digital agents [61, 63, 72]. Similar findings have been reported in digital mental health contexts, where users with greater distress often report higher perceived empathy and trust in chatbot-based support tools [30, 73]. Moreover, research specifically examining technology use among foster care populations indicates that digital platforms may offer a sense of privacy and agency that is particularly valued by those who have experienced institutional oversight and limited control over their lives [57, 60, 74].

Conversely, users who cited convenience as their primary reason for chatbot use, or who rated themselves highly proficient with smartphones, were significantly less likely to perceive the chatbot as trauma-informed. This pattern aligns with prior work suggesting that technologically experienced or efficiency-oriented users tend to evaluate conversational systems through a utilitarian lens, prioritizing speed, task completion, and functional accuracy over relational or emotional qualities [75, 76]. Additionally, higher smartphone proficiency may foster greater critical awareness of automated empathy, leading some users to approach relational or caring language with increased skepticism [77]. Related work in digital self-care and health technologies has similarly shown that users motivated by convenience often place lower value on emotional depth, which can lead to reduced perceptions of empathy or trust [54]. In such cases, trauma-informed features, such as validation, reflective language, or user control, may be perceived as peripheral or insufficient relative to expectations for seamless performance.

Overall, our results offer critical insights for the future of trauma-informed AI in mental health. First, we demonstrate that TIC is both legible and empirically measurable: users overwhelmingly endorsed trauma-informed experiences, and factor analysis revealed a robust five-domain structure that provides strong evidence for measurement accuracy. Second, predictive modeling identified high-impact predictors of trauma-informed perceptions, spanning both chatbot design features (e.g., trust, empowerment, and peer support) and user characteristics (e.g., trauma history, motivations for use, and technology proficiency), thereby underscoring the importance of personalization and user-centered design. Third, our findings reveal meaningful variation in how different user populations perceive and prioritize TIC features, suggesting that one-size-fits-all approaches may inadequately serve diverse trauma survivors. Designers of mental health chatbots should embed trauma-informed principles not as optional enhancements but as core architectural features. Likewise, TIC-aligned metrics should be systematically incorporated into evaluation frameworks. As reliance on automated tools for mental health support continues to grow, establishing rigorous standards for safety, ethics, and human-centered care will be essential.

Several limitations should be considered when interpreting these findings. First, the study employed a cross-sectional, self-reported survey design, which may be influenced by recall bias or social desirability effects. Although the sample was demographically diverse, it was predominantly White and limited to English-speaking individuals with reliable internet access, potentially restricting generalizability to more diverse or digitally marginalized populations. Future research should intentionally oversample underrepresented groups, including racial and ethnic minorities, non-English speakers, and individuals with limited digital access, to ensure equity in TIC design and evaluation [78]. Second, despite multiple attempts to recruit through the MyChart patient portal, only five participants were enrolled via this channel, limiting meaningful comparisons across recruitment sources and underrepresenting clinical populations. Third, while the regression results highlight meaningful associations between trauma-informed perception and both chatbot design features and user characteristics, the high prevalence of positive outcomes introduces ceiling effects that may inflate odds ratios and reduce estimate stability for rare subgroups. Accordingly, these findings should be interpreted as identifying salient patterns and hypotheses for future validation in more balanced and clinically diverse samples. Finally, although the study relied primarily on quantitative survey data, which provides breadth, it offers limited depth regarding lived experiences. To strengthen data triangulation, the research team is conducting complementary semi-structured interviews as part of the broader project.

In conclusion, our study establishes a rigorous empirical foundation for understanding how TIC principles manifest in mental health chatbot interactions. By integrating descriptive analysis, factor modeling, predictive regression, and qualitative insights, we demonstrate that TIC is both measurable and impactful, with trust, empowerment, and peer support emerging as central drivers of user perceptions alongside key individual characteristics. To advance trauma-informed digital mental health, developers should prioritize hybrid models that integrate peer networks and human–bot collaboration, tailor functionality to diverse user profiles, and systematically evaluate emerging TIC dimensions. Future longitudinal and multi-platform research will be critical for validating and extending these findings, ultimately guiding the design of more inclusive, effective, and psychologically safe chatbot interventions. As artificial intelligence becomes increasingly embedded in mental health service delivery, the ethical imperative to center trauma survivors’ voices, protect their safety, and honor their agency has never been more urgent [79]. This study represents an essential step toward realizing that vision.

## Data Availability

The original contributions presented in the study are publicly available. This data can be found here: https://gitlab.com/E190153/user-perceptions-of-trauma-informed-chatbots.
