# Supplementary material for: Trauma-informed conversational agents for mental health: understanding user perspectives and experiences
Source: Front Digit Health. 2026 Jun 10;8:1797681. doi: 10.3389/fdgth.2026.1797681 (PMC13291057; doi:10.3389/fdgth.2026.1797681)
Supplement: Supplementary file 1 [file Supplementaryfile1.pdf]

Supplementary Material

MENTAL HEALTH CHATBOT USER SURVEY

Survey Questions

Demographics

Q1. What is your gender?

- Female
- Male
- Transgender
- Nonbinary
- Other (please specify): ____________________
- Prefer not to say

Q2. What is your age?

- 18–24 years old
- 25–34 years old
- 35–44 years old
- 45–54 years old
- 55–64 years old
- 65 years or older

Q3. What is your race? (Select all that apply)

- American Indian or Alaska Native
- Asian
- Black or African American
- Native Hawaiian or Other Pacific Islander
- White
- Other (please specify): ____________________

Q4. Select which of the following you have experienced in life? (Select all that apply)

- Abuse (e.g., physical, sexual, emotional)
- Neglect (e.g., physical, emotional)
- Household Dysfunction (e.g., substance abuse in the household, mental illness in the household, domestic violence, parental separation/divorce, incarceration of a family member)
- Community-Related Adverse Experiences (e.g., physical bullying, cyberbullying, natural disaster, discrimination)
- Adoption

- Involvement with Department of Child Services

Q5. What is your highest level of education?

- Less than High School
- High school (including GED)
- Some college (no degree)
- Technical certification
- Associate degree (2-year)
- Bachelor's degree (4-year)
- Master's degree
- Doctoral degree
- Professional degree (JD, MD)
- Prefer not to say

Technology Experience

Q6. How good or proficient do you think you are at using different technologies? *(1 = Not good/proficient, 5 = Very good/proficient)*

• Smart phones	1 2 3 4 5
• Browsing internet	1 2 3 4 5
• AI Tools (e.g., Chatbots, ChatGPT, Woebot, Wysa)	1 2 3 4 5
• Desktop or Laptop Computers	1 2 3 4 5
• Social Media Platforms (e.g., Facebook, Twitter)	1 2 3 4 5
• Smart Home Devices (e.g., Amazon Alexa, Google Home)	1 2 3 4 5

Q7. Which mental health chatbot(s) have you used? (Select all that apply)

- Wysa
- Woebot
- Youper
- Moodkits
- ADA
- Chai
- Elomia
- Mindspa
- Nuna
- Serenity
- Stresscoach
- Others

Q7a. Please specify which other chatbot you have used (#1): ______________________________

Q7b. Please specify which other chatbot you have used (#2): ______________________________

Q8. Why did you use a mental health based chatbot(s)? (Select all that apply)

- Seeking support for managing stress and anxiety
- Coping with depression or low mood
- Dealing with loneliness or social isolation
- Coping with grief/loss
- Coping with trauma
- Exploring new ways to help with my mental health
- To have a safe space to express my thoughts and feelings without judgment
- Recommendation from a friend, family member, or healthcare professional
- Curious about mental health chatbots
- Previous positive experiences with similar apps or services
- Convenience and accessibility compared to traditional therapy
- Preference for privacy and/or anonymity
- Tracking mood and progress over time
- Easy access to mental health resources (including peer support)
- Others

Q8a. Please specify other reasons (#1): ______________________________

Q8b. Please specify other reasons (#2): ______________________________

Q9. How frequently do you use mental health chatbot(s)?

- Multiple times per day
- Once daily
- 2–5 times per week
- Once per week
- 1–3 times per month
- Less than once a month
- I only used the app to try it out

Q10. Did you face any problems while using the mental health chatbot(s)? *(Open-ended)*

Trauma-Informed Questions

If you have used multiple mental health chatbots, please select one while answering the questions below.

Selected chatbot: ______________________________

Safety

Q11. To you, feeling safe while using or chatting with mental health chatbots means feeling... *(Select all that apply)*

- Physically safe
- Emotionally/mentally safe

- Digitally safe
- None of the above
- Other forms of safety

Q11a. Please specify other types of safety (#1): ______________________________

Q11b. Please specify other types of safety (#2): ______________________________

Q12–Q16. Please indicate how strongly you agree or disagree *(1 = Never, 5 = Always)*

- Q12. Physical safety 1 2 3 4 5
- Q13. Emotional/mental safety 1 2 3 4 5
- Q14. Digital safety 1 2 3 4 5
- Q15. Privacy and confidentiality 1 2 3 4 5

Q16. Design features or conversational aspects that made you feel safer *(Open-ended)*

Transparency / Trustworthiness

Q17. To you, a trustworthy or transparent mental health chatbot means... *(Select all that apply)*

- Does not share personal information without consent
- Validates emotions and concerns
- Provides tailored (non-generic) responses
- Is transparent about capabilities and limitations
- Responds in ways that meet expectations
- Is reliable and always available
- Offers helpful suggestions
- Handles personal data with care and consent
- Others

Q17a. Please specify other ways chatbots can be trustworthy/transparent: ______________________________

Q18–Q25. Please indicate how strongly you agree or disagree *(1 = Strongly Disagree, 5 = Strongly Agree)*

- Q18. Information confidentiality 1 2 3 4 5
- Q19. Validates experiences 1 2 3 4 5
- Q20. Tailored responses 1 2 3 4 5
- Q21. Transparency 1 2 3 4 5
- Q22. Meets expectations 1 2 3 4 5
- Q23. Reliability 1 2 3 4 5
- Q24. Helpful suggestions 1 2 3 4 5
- Q25. Data care 1 2 3 4 5

Q26. Design features that helped you trust the chatbot more *(Open-ended)*

BOT Honeypot: What is your favorite color? ____________________

Empowerment / Voice / Choice

Q27. Being in control or feeling empowered means… *(Select all that apply)*

- Guide or direct the conversation
- Control how experiences are shared
- Not feeling forced to select options
- Wide range of inclusive options
- Fosters hope
- Builds sense of agency
- Others

Q27a. Please specify other empowerment features: ______________________________

Q28–Q35. Please indicate agreement *(1 = Strongly Disagree, 5 = Strongly Agree)*

• Q28. Guide conversation	1 2 3 4 5
• Q29. Attention check	1 2 3 4 5
• Q30. Sharing control	1 2 3 4 5
• Q31. No forced choice	1 2 3 4 5
• Q32. Expression options	1 2 3 4 5
• Q33. Hopeful messaging	1 2 3 4 5
• Q34. Sense of agency	1 2 3 4 5

Q35. Design features that increased empowerment *(Open-ended)*

Collaboration

Q36. Collaboration means… *(Select all that apply)*

- Setting goals collaboratively
- Respecting opinions and choices
- Working on shared tasks and goals
- Others

Q36a. Please specify other collaboration features: ______________________________

Q37–Q39. Please indicate agreement *(1 = Strongly Disagree, 5 = Strongly Agree)*

• Q37. Goal setting	1 2 3 4 5
• Q38. Respect choices	1 2 3 4 5
• Q39. Shared goals	1 2 3 4 5

Q40. Design features supporting collaboration *(Open-ended)*

Peer Support

Q41. Peer support means… *(Select all that apply)*

- Receiving peer support

- Providing peer support
- Asking for peer support
- Relevant connections
- Virtual and in-person referrals
- Others

Q41a. Please specify other peer support features: ______________________________

BOT Honeypot: Favorite ice-cream flavor

- Vanilla
- Chocolate
- Butter pecan
- Coconut
- Others

Q42–Q46. Please indicate agreement *(1 = Strongly Disagree, 5 = Strongly Agree)*

- Q42. Support available 1 2 3 4 5
- Q43. Offer support 1 2 3 4 5
- Q44. Ask for support 1 2 3 4 5
- Q45. Relevant connections 1 2 3 4 5
- Q46. Virtual/In-person support 1 2 3 4 5

Q47. Design features supporting peer support *(Open-ended)*

Cultural / Gender / Historical Sensitivity

Q48. Sensitivity means… *(Select all that apply)*

- Sensitive to identity and experiences
- Avoids offensive language
- Avoids triggers
- Nonjudgmental
- Validates experiences
- Shows interest
- Culturally aligned responses
- Others

Q48a. Please specify other sensitivity features: ______________________________

Q49–Q55. Please indicate agreement *(1 = Strongly Disagree, 5 = Strongly Agree)*

- Q49. Sensitive to experiences 1 2 3 4 5
- Q50. Avoids offense 1 2 3 4 5
- Q51. Avoids triggers 1 2 3 4 5
- Q52. Nonjudgmental 1 2 3 4 5

- Q53. Validates experiences 1 2 3 4 5
- Q54. Shows interest 1 2 3 4 5
- Q55. Culturally aligned 1 2 3 4 5

Q56. Design features demonstrating sensitivity *(Open-ended)*

Satisfaction

Q57. Overall satisfaction *(1 = Extremely dissatisfied, 5 = Extremely satisfied)* 1 2 3 4 5

Q58. Is your chatbot trauma-informed?

- Yes
- No

Q59. Would you recommend this chatbot to someone with trauma?

- Yes
- No

Q60. Follow-up participation

- I want to participate in a follow-up interview
- I want to get compensated

Q60a. If selected, please provide your email address: ________________________________
