# Supplementary material for: Trauma-informed conversational agents for mental health: understanding user perspectives and experiences
Source: Front Digit Health. 2026 Jun 10;8:1797681. doi: 10.3389/fdgth.2026.1797681 (PMC13291057; doi:10.3389/fdgth.2026.1797681)
Supplement: Supplementary file 2 [file Supplementaryfile2.pdf]

Supplementary Material

1 SUPPLEMENTARY FIGURES

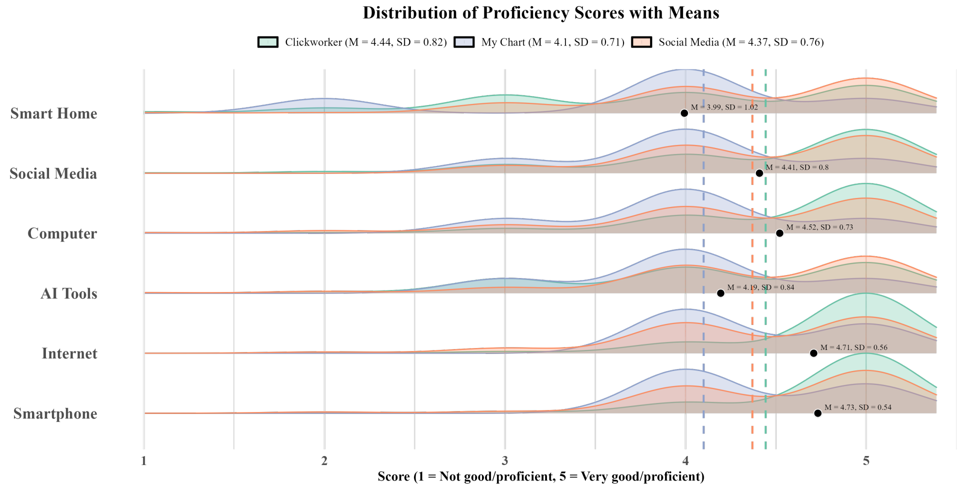

Figure S1. Distribution of Proficiency Scores with Means. Vertical dashed lines represent group-specific means for each collection type. Black dots indicate the overall sample mean for each domain.

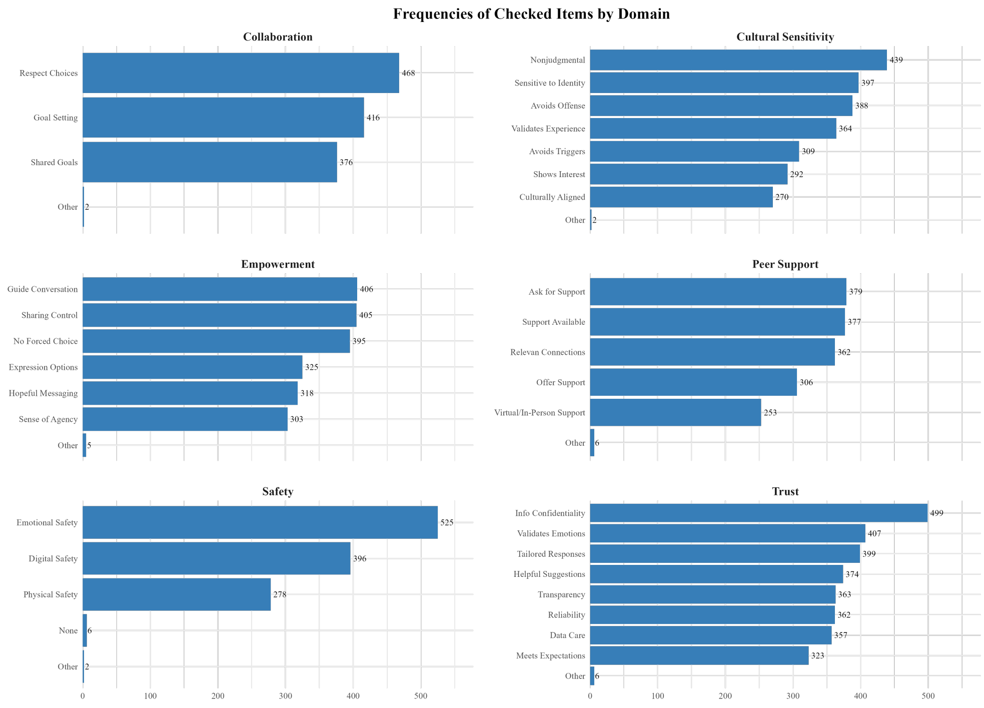

Figure S2. Frequency of participant-selected trauma-informed chatbot features across six core domains (N = 606).

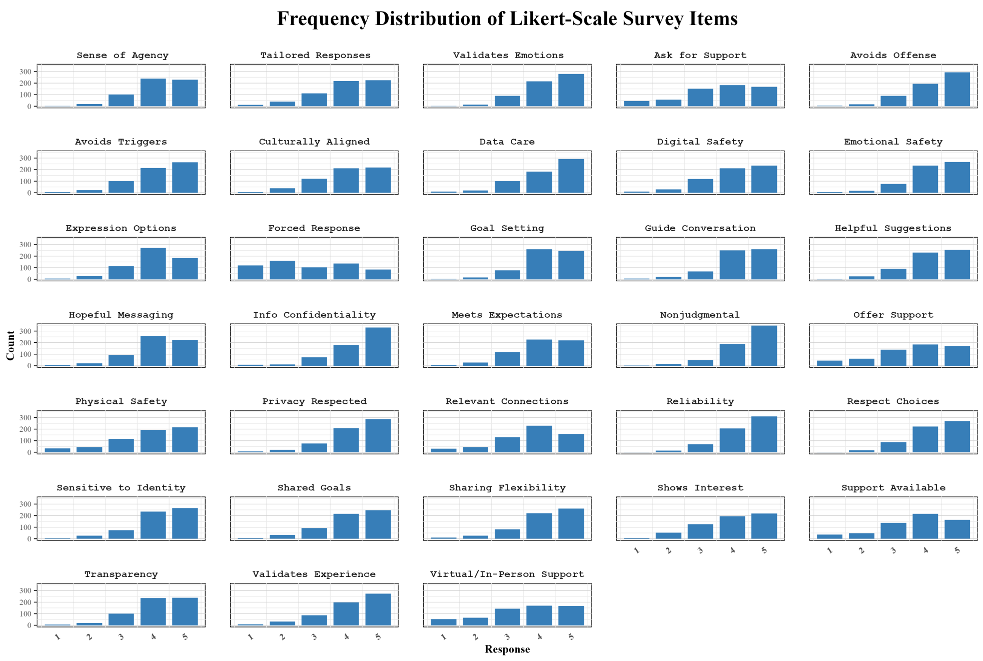

Figure S3. Distribution of Likert-Scale Responses Across 33 Trauma-Informed Care Survey Item.

2 SUPPLEMENTARY TABLES

Table S1. Overview of Survey Content by Section

Survey Structure and Measurement Design			
**Section**	**Items**	**Example Question**	**Response Type**
Demographics	Gender, age, race/ethnicity, education level, prior adverse life experiences	Select which of the following you have experienced in life?	Multiple-response
Technology Experience	Smartphone use, AI tools, social media, smart devices	How proficient are you at using AI tools (e.g., ChatGPT, Woebot)?	Likert scale (1–5)
	Frequency of use, specific chatbot(s) used, reasons for use (e.g., depression, privacy, convenience)	Why did you use mental health–based chatbot(s)?	Multiple-response; open-ended
Trauma-Informed Questions	Perceptions and experiences related to safety, trustworthiness/transparency, empowerment, voice/choice, collaboration, peer support, and cultural sensitivity	To you, feeling safe while using or chatting with mental health chatbots means feeling. . .	Multiple-response
	Physical safety during vulnerable disclosure	To what extent did/does your chatbot ensure your physical safety when you share vulnerable thoughts or experiences?	Likert scale (1–5); open-ended
Satisfaction & Perceptions	Overall satisfaction, trauma-informed perception, willingness to recommend	Overall, based on your experience, do you think your chatbot is trauma-informed?	Likert scale; binary (Yes/No)

Table S2. Overview of Survey Content of the TIC Section and its Short Label

Trauma-Informed Care Survey Item Mapping		
**Multiple Response Section**	**Likert Section**	**Label**
**Safety**		
Physically safe (e.g., chatbot ensures your physical safety if you are in an unsafe situation)	12. To what extent did/does your chatbot ensure your physical safety when you share vulnerable thoughts or experiences?	Physical Safety
Emotionally/mentally safe (e.g., you can share anything with the chatbot without any fear or judgement)	13. To what extent did/does your chatbot ensure your emotional/mental safety when you share vulnerable thoughts or experiences?	Emotional Safety
Digitally safe (e.g., information you share with chatbot is protected and private)	14. To what extent did/does your chatbot ensure your digital safety when you share vulnerable thoughts or experiences?	Digital Safety
	My mental health chatbot respects my privacy and confidentiality during interactions.	Privacy Respected
**Trust and Transparency**		
A tool that does not share your personal information outside without your consent	18. My chatbot does not share my personal information with anyone without my consent	Info Confidentiality
A tool that validates/acknowledges your emotion and concerns	19. My experiences and concerns are always acknowledged and/or validated by my chatbot	Validates Experiences
A tool that provides tailored response to your concern (e.g., not repetitive, generic)	20. My chatbot's responses are tailored based on what I share with it and not generic	Tailored Responses
A tool that is transparent to you about its capabilities and limitations in supporting you	21. My chatbot is transparent with me about its capabilities and limitations in supporting me	Transparency
A tool that responds to your experiences in a way that meets your expectations	22. My chatbot responds to my experiences in a way that meets my expectations	Meets Expectations
A tool that is reliable and always available to support you whenever you need it	23. My chatbot is reliable and always available to support me whenever I need it	Reliability
A tool that offers helpful suggestions whenever you need it	24. My chatbot offers helpful suggestions whenever I need it	Helpful Suggestions
A tool that handles your personal information and data with utmost care and consent	25. My mental health chatbot handles my personal information and data with care and does not share my information outside without my consent.	Data Care
**Empowerment**		
Having the ability to guide or direct the conversation with chatbot as you see fit	28. I have the ability to guide or direct the conversation with chatbot as I see fit	Guide Conversation
Being in control how you share your experiences and feelings with chatbot (e.g., using video, audio, or chat options)	30. I decide how I want to share my experiences or feelings with the chatbot either through text, audio, or video. Chatbot does not limit me	Sharing Control
Not feeling forced to select an option offered by the chatbot	31. I sometimes feel forced to choose a specific response type or option suggested by my chatbot, which limits my ability to express myself freely	No Forced Choice
Having a wide range of inclusive options from the chatbot during discussions	32. My chatbot provides a wide range of options during conversations (e.g., not restricting within just yes/no) that help me to express myself better	Expression Options
Having a chatbot that fosters hope and belief that things can improve for you	33. My chatbot helps me believe that there is hope and things can improve for me	Hopeful Messaging
Having a chatbot that helps you believe that you have agency/ability to change things in your life	34. My chatbot helps me believe that I have agency/ability to change things in my life	Sense of Agency
**Collaboration**		
Setting your own mental health goals or strategies through mutual discussion with the chatbot	37. I can set my own mental health goals or strategies through mutual discussion with my chatbot	Goal Setting
Your opinions and choices are respected and considered as chatbot creates support strategies and treatment recommendations	38. My opinions and choices are respected and considered as chatbot creates support strategies and treatment recommendations	Respect Choices
Feeling confident that the chatbot and you are working on shared tasks and goals	39. I feel confident that the chatbot and I are working on shared tasks and goals	Shared Goals

Trauma-Informed Care Survey Item Mapping		
**Multiple Response Section**	**Likert Section**	**Label**
**Peer Support**		
Having opportunities to receive peer support or community connection through chatbot	42. My mental health chatbot offers opportunities for peer support and community connections as part of my mental health management	Support Available
Having opportunities to provide peer support or community connection to others through chatbot	43. My mental health chatbot gives me opportunities to offer peer support and community connections to others struggling with similar experiences	Offer Support
Having opportunities to ask for peer support/community connections/referrals through chatbot	44. I always have opportunities to ask for peer support/community connections/referrals through my chatbot	Ask for Support
Having relevant and helpful peer support and/or community connection opportunities through chatbot	45. My chatbot shares relevant and helpful peer support and/or community connection opportunities for me	Relevant Connections
Having both virtual and in-person peer support referrals (whenever relevant) through chatbot	46. My chatbot provides me both virtual and in-person peer support referrals (whenever relevant)	Virtual/In-Person Support
**Cultural Sensitivity**		
Chatbot is sensitive to your concerns, identity, and/or historical experiences	49. My chatbot is sensitive to my concerns, identity, and/or historical experiences	Sensitive to Identity
Chatbot avoids words or phrases that may be offensive or minimizes your experiences	50. My chatbot avoids using words or phrases that may be offensive or minimizes my experiences	Avoids Offense
Chatbot avoids words or phrases that trigger you	51. My chatbot avoids words or phrases that may trigger me	Avoids Triggers
Chatbot does not judge or criticize you when you share your sensitive experiences	52. My chatbot does not judge or criticize me when I share my sensitive experiences	Nonjudgmental
Chatbot validates your feelings and experiences related to your cultural, gender or historical background	53. My chatbot validates my feelings and experiences related to my cultural, gender or historical background	Validates Experience
Chatbot shows interest to learn more about your unique cultural, gender or historical background	54. My chatbot shows interest to learn more about my unique cultural, gender or historical background	Shows Interest
Chatbot offers responses, suggestions or resources that are aligned with your cultural, gender or historical preferences	55. My chatbot offers responses, suggestions or resources that are aligned with my cultural, gender or historical preferences	Culturally Aligned

Table S3. Exploratory Factor Analysis Results with Communality and Standardized Factor Loadings

Factor and Item	Communality	Standardized Loading
**Trust**		
Q19. Validates Experiences	0.59	0.61
Q20. Tailored Responses	0.64	0.70
Q21. Transparency	0.50	0.52
Q22. Meets Expectations	0.66	0.75
Q23. Reliability	0.45	0.61
Q24. Helpful Suggestions	0.59	0.77
**Safety**		
Q14. Digital Safety	0.51	0.69
Q15. Privacy Respected	0.76	0.87
Q25. Data Care	0.65	0.66
**Empowerment**		
Q33. Hopeful Messaging	0.57	0.58
Q34. Sense of Agency	0.99	1.00
**Peer Support**		
Q42. Support Available	0.79	0.86
Q43. Offer Support	0.73	0.87
Q44. Ask for Support	0.69	0.78
Q45. Relevant Connections	0.73	0.83
Q46. Virtual/In-Person Support	0.65	0.81
**Cultural Sensitivity**		
Q50. Avoids Offense	0.88	0.93
Q51. Avoids Triggers	0.50	0.51

Table S4. Confirmatory Factor Analysis Results with Standardized Factor Loading, Squared Multiple Correlation, Average Variance Extracted and Construct Reliability.

Factor and Item	Std. Loading	SMC	AVE[a]	CR[b]
**Trust**			0.563	0.885
Q19. Validates Experiences	0.773	0.60		
Q20. Tailored Responses	0.800	0.64		
Q21. Transparency	0.713	0.51		
Q22. Meets Expectations	0.805	0.65		
Q23. Reliability	0.644	0.41		
Q24. Helpful Suggestions	0.754	0.57		
**Safety**			0.629	0.835
Q14. Digital Safety	0.699	0.49		
Q15. Privacy Respected	0.825	0.68		
Q25. Data Care	0.847	0.72		
**Empowerment**			0.731	0.844
Q33. Hopeful Messaging	0.825	0.68		
Q34. Sense of Agency	0.884	0.78		
**Peer Support**			0.721	0.928
Q42. Support Available	0.883	0.78		
Q43. Offer Support	0.855	0.73		
Q44. Ask for Support	0.839	0.70		
Q45. Relevant Connections	0.857	0.73		
Q46. Virtual/In-Person Support	0.811	0.66		
**Cultural Sensitivity**			0.614	0.760
Q50. Avoids Offense	0.758	0.57		
Q51. Avoids Triggers	0.808	0.65		

[a]AVE: Average Variance Extracted. [b]CR: Construct Reliability.
